# Identification of Cross-Talk and Pyroptosis-Related Genes Linking Periodontitis and Rheumatoid Arthritis Revealed by Transcriptomic Analysis

**DOI:** 10.1155/2021/5074305

**Published:** 2021-12-29

**Authors:** Yongbin Jing, Dong Han, Chunyang Xi, Jinglong Yan, Jinpeng Zhuang

**Affiliations:** ^1^Department of Orthopeadics, The 2nd Affiliated Hospital of Harbin Medical University, 246 Xuefu Road, Harbin 150081, China; ^2^Department of Human Movement and Sport Science, Harbin Sport University, 1 Dacheng Street, Nangang District, Harbin 150008, China

## Abstract

**Background:**

The current study is aimed at identifying the cross-talk genes between periodontitis (PD) and rheumatoid arthritis (RA), as well as the potential relationship between cross-talk genes and pyroptosis-related genes.

**Methods:**

Datasets for the PD (GSE106090, GSE10334, GSE16134) and RA (GSE55235, GSE55457, GSE77298, and GSE1919) were downloaded from the GEO database. After batch correction and normalization of datasets, differential expression analysis was performed to identify the differentially expressed genes (DEGs). The cross-talk genes linking PD and RA were obtained by overlapping the DEGs dysregulated in PD and DEGs dysregulated in RA. Genes involved in pyroptosis were summarized by reviewing literatures, and the correlation between pyroptosis genes and cross-talk genes was investigated by Pearson correlation coefficient. Furthermore, the weighted gene coexpression network analysis (WGCNA) was carried out to identify the significant modules which contained both cross-talk genes and pyroptosis genes in both PD data and RA data. Thus, the core cross-talk genes were identified from the significant modules. Receiver-operating characteristic (ROC) curve analysis was performed to identify the predictive accuracy of these core cross-talk genes in diagnosing PD and RA. Based on the core cross-talk genes, the experimentally validated protein-protein interaction (PPI) and gene-pathway network were constructed.

**Results:**

A total of 40 cross-talk genes were obtained. Most of the pyroptosis genes were not differentially expressed in disease and normal samples. By selecting the modules containing both cross-talk genes or pyroptosis genes, the blue module was identified to be significant module. Three genes, i.e., cross-talk genes (TIMP1, LGALS1) and pyroptosis gene-GPX4, existed in the blue module of PD network, while two genes (i.e., cross-talk gene-VOPP1 and pyroptosis gene-AIM2) existed in the blue module of RA network. ROC curve analysis showed that three genes (TIMP1, VOPP1, and AIM2) had better predictive accuracy in diagnosing disease compared with the other two genes (LGALS1 and GPX4).

**Conclusions:**

This study revealed shared mechanisms between RA and PD based on cross-talk and pyroptosis genes, supporting the relationship between the two diseases. Thereby, five modular genes (TIMP1, LGALS1, GPX4, VOPP1, and AIM2) could be of relevance and might serve as potential biomarkers. These findings are a basis for future research in the field.

## 1. Introduction

The relationship between rheumatoid arthritis (RA) and periodontitis (PD) was extensively examined during recent years; a systematic review and meta-analysis revealed that patients with RA had a 1.69-fold increased risk of PD, albeit a certain heterogeneity is given across studies [1]. Altogether, it is meanwhile well-established that RA and PD are similar diseases, whereby main similarities are a chronic inflammatory character driven by proinflammatory cytokines and leading to destruction of hard and soft tissues, combined with shared risk factors, e.g., smoking and ageing [2]. While the primary biological correlation between these two diseases is founded on three mechanisms, i.e., genetic susceptibility, microbial status, and (auto-)inflammatory response, the detailed mechanisms are still not fully understood [3].

Especially, the transfer of findings originating from animal models into clinical context appears limited; thereby, the respective validation in patient cohorts is often difficult. To outline one example, the citrullination of autoantibodies by *Porphyromonas gingivalis*, a potentially pathogenic, gram-negative anaerobic bacterium, has been repeatedly proven in an animal model [4]. However, large clinical cohort studies were not able to confirm these findings completely, showing contradictory results [4–6]. Due to the variety of potential interactive pathways between PD and RA, the heterogeneity across different studies and the incomplete knowledge on the role of proinflammatory cytokines, more research is needed to gain a deeper understanding of the link between these two diseases [4, 7]. In this context, the inflammasome and pyroptosis could be a promising new approach; pyroptosis is a proinflammatory form of cell death, which is characterized by membrane pore formation, rapid swelling, and lysis of respective cells, alongside with an emerging release of proinflammatory mediators [8, 9]. Aside of apoptosis and necroptosis, pyroptosis is one out of the three most well-understood modalities of cell death. Moreover, it plays an important role in autoimmunity [10]. Accordingly, it is not surprising that pyroptosis is of relevance in RA pathogenesis, especially due to the link with plasma membrane destruction and release of proinflammatory mediators [11]. Thereby, cell death has been developed into a field of growing interest in autoimmunity, including understanding of pathogenesis as well as finding appropriate therapeutic targets [12]. Up until now, no examination of pyroptosis in the interrelationship between PD and RA has been performed.

Therefore, this current study is aimed at evaluating cross-talk genes between PD and RA and their potential relationship with pyroptosis-related genes to reveal their potential role in the interrelationship between these two diseases. For this, bioinformatics analysis was applied to allow the generation of respective hypothesis as a basis for future clinical research in the field. The analysis included assessment of overlapping genes dysregulated in PD and RA, which are hypothesized to be key players in the interrelationship between these two diseases. Moreover, a potential correlation between pyroptosis-related genes and the identified cross-talk genes should be examined to identify the role of pyroptosis in the interplay between PD and RA. Due to the identification of biological processes and pathways in this interrelationship, a deeper understanding of the shared genetic mechanisms between PD and RA should be gained. The general hypothesis of this current study was that there would be a couple of pyroptosis-related genes, which are related to the genetic cross-talk between PD and RA on transcriptomic level.

## 2. Material and Methods

### 2.1. The Work Flowchart of the Current Research

**Figure 1:**
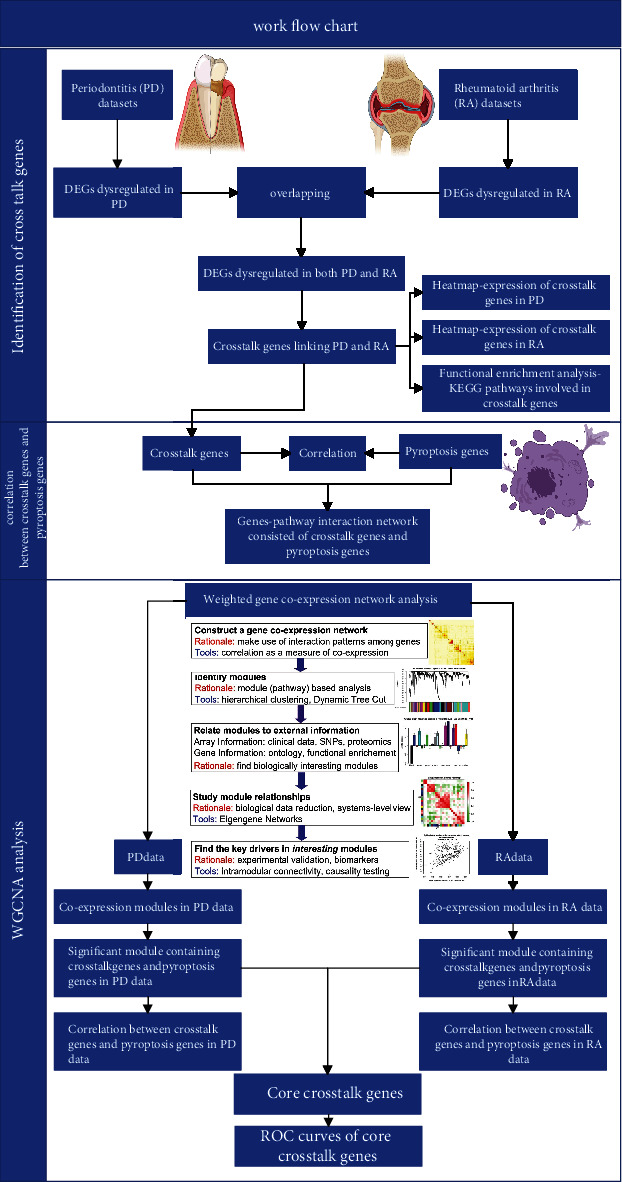
The work flowchart of the current study.

Figure 1 illustrated the study design of the current research. As shown in Figure 1, the bioinformatics analysis consisted of three steps. Firstly, identification of cross-talk genes linking PD and RA, which were obtained by overlapping the DEGs dysregulated in PD and DEGs dysregulated in RA. Secondly, genes involved in pyroptosis were summarized by reviewing literatures, and the correlation between pyroptosis genes and cross-talk genes was investigated by using correlation heatmap. Furthermore, the weighted gene coexpression network analysis (WGCNA) was carried out to identify the significant modules which contained both cross-talk genes and pyroptosis genes in both PD data and RA data. Thus, the core cross-talk genes were identified from the significant modules.

### 2.2. Data Procurement and Preprocessing

Sample-matched whole-genome gene expression datasets from periodontitis were sourced and downloaded from the Gene Expression Omnibus (GEO) [13] in National Center for Biotechnology Information (NCBI) [14]. The eligibility criteria for these datasets were as follows: datasets that included established periodontitis samples as the experimental group and healthy gingival samples as the control group, where periodontitis was defined based in accordance with the case-definition presented in the 2017 world workshop on the classification of periodontal and peri-implant diseases and conditions: (1) interdental CAL detectable at ≥2 nonadjacent teeth or (2) buccal or oral CAL ≥3 mm with pocketing >3 mm detectable at ≥2 teeth [15]. Based on such inclusion criterion, three periodontitis-related datasets (i.e., GSE106090 [16], GSE10334 [17], and GSE16134 [18, 19]) were included in the current analysis.

Regarding rheumatoid arthritis (RA), the datasets with the established study design of comparing the genetic expression alteration of the synovial tissue between RA patients and non-RA subjects were included; thus, four datasets (i.e., GSE55235 [20], GSE55457 [20], GSE77298 [21], and GSE1919 [22]) were assessed from the GEO database. The experimental type of all included datasets for both diseases was consistent: all datasets were microarray datasets. The probe IDs in the datasets were converted into gene symbol by comparing the information in the relevant experimental platform with the Homo sapiens (human) genome assembly GRCh37 (hg19). The detailed information (e.g., sample types, experimental type, experimental platform, as well as the number of case and control samples) of each included GEO dataset is shown in Table 1. The detailed clinical sample information of each dataset is shown in the supplementary material Table S1.

### 2.3. Batch Correction and Normalization of PD and RA Data

Based on the information of the datasets (Table 1), the intersection genes between datasets of each type of disease were obtained. In other words, regarding PD, the overlapping between genes examined in GSE106090, GSE10334, and GSE16134 datasets was obtained, and regarding RA, the overlapping between genes examined in GSE55235, GSE55457, GSE77298, and GSE1919 was obtained. The expression profiles of these intersection genes in each dataset were obtained, respectively. Afterward, all samples for each type of disease were combined based on the expression profiles of the intersection genes in each dataset.

In order to reduce the deviation caused by the merging process of combining different batches of samples, the ComBat method in the sva package (version 3.13) of R program (version 3.6.3) was used to perform batch correction of merged data [23]. Since the expression value of RA samples were comparatively larger than that of PD samples, the expression profile of RA data was firstly corrected and then normalized by using scale() function of R program (version 3.6.3).

### 2.4. Differential Expression Analysis

After batching correction, the differential expression analysis (DEA) was performed on the corrected PD and RA data by using limma package (version 3.9) of R program (version 3.6.3) [24]. The genes with *P* value < 0.05 and ∣logFC | > = 0.5 were defined as differentially expressed genes (DEGs). By performing DEA analysis, DEGs which were dysregulated in RA and PD (i.e., RA-DEGs and PD-DEGs) were identified, respectively. The expression pattern of DEGs identified in RA and PD was depicted in the volcano plot by using the ggplot2 package (version 3.3.5) of R program (version 3.6.3).

### 2.5. Cross-Talk Genes Linking PD and RA

The evaluation of cross-talk genes was a major aim of this current study. Thereby, cross-talk genes were defined as genes, which were jointly dysregulated in both RA and PD. Those genes are supposed to be key players in the interrelation between PD and RA by regulating biological processes or pathways, which are important for RA patients to develop more severe PD (and vice versa). After identifying the DEGs in PD and RA, respectively, the intersection between RA-DEGs and PD-DEGs were obtained. Such intersection genes were not only differentially expressed in RA but also differentially expressed in PD; thus, these genes were regarded as cross-talk genes linking PD and RA. The expression patterns of the cross-talk genes in diseased and healthy control samples of PD and RA were visualized by heatmap, using ggplot2 package (version 3.3.5) of R program (version 3.6.3). The functional enrichment analysis was performed to identify the biological functions of cross-talk genes, especially from the aspect of biological processes and signaling pathway.

### 2.6. Correlation between Cross-Talk Genes and Pyroptosis-Related Genes

In order to investigate the role of pyroptosis in regulating the linkage between PD and RA, the correlation between cross-talk genes and pyroptosis-related genes was analyzed. A total of 33 pyroptosis-related genes (Table 2) were obtained by reviewing previous literature regarding pyroptosis [25–28].

The expression values of pyroptosis-related genes and cross-talk genes in case and control samples of PD and RA were obtained, respectively. The case samples of PD were gingival tissue of periodontitis patients, while the control samples of PD data were gingival tissue of periodontal healthy subjects. The case samples of RA data were synovial tissue from osteoarthritic joint, while the control samples of RA data were synovial tissue from healthy joint. Subsequently, the correlation between pyroptosis genes and cross-talk genes in different types of samples was analyzed by performing the Pearson correlation method. The Pearson correlation coefficient (*r*) values were calculated by using the “corrplot” package (version 0.90) [29] of R program (version 3.6.3).

### 2.7. The Functional Relationship between Cross-Talk Genes and Pyroptosis Genes

The functional relationship between cross-talk genes and pyroptosis-related genes was evaluated. The human KEGG pathways and pathway-gene datasets were obtained from the KEGG database (https://www.kegg.jp/) [30]. The common pathways regulated by both cross-talk genes and pyroptosis-related genes were obtained from pathway-gene datasets. Such pathways were the key pathways by which the cross-talk genes and pyroptosis-related genes act together. The gene-pathway network consisted of the cross-talk genes, and pyroptosis genes were visualized by using Cytoscape software (version 3.9.0) [31].

### 2.8. Weighted Gene Coexpression Network Analysis

The weighted gene coexpression network analysis (WGCNA) was performed to identify the significant modules that contain both cross-talk genes and pyroptosis-related genes. The gene coexpression networks were constructed based on case samples of PD and RA data, respectively. Firstly, the expression values of all genes in the PD and RA datasets were obtained, respectively. Afterward, the “wgcna” package (version: 1.70-3) [32] of R program (version 3.6.3) was used to screen the coexpressed modules, respectively, belonging to PD and RA data. The coexpression network was constructed, and coexpression gene modules were further identified. The methodology of WGCNA analysis followed the flowchart described by Langfelder and Horvath [33].

Briefly, WGCNA analysis follows several steps as below: firstly, an unsupervised coexpression relationship was initially built based on the adjacency matrix of connection strengths by using Pearson's correlation coefficients for gene pairs. The power *β* was calculated, using the “pickSoftThreshold” function. The arguments (corFnc=“bicor,” corOptions=list (maxPOutliers=0.1), network type=“signed,” power=“*β*”) were chosen to meet the need of scale-free topology property of the coexpression network. Based on the scale-free topology criterion, the optimum power *β* was selected to amplify the strong connections between genes and penalize the weaker connections. Furthermore, the hybrid dynamic tree cutting method was used to cut branches and cluster coexpression modules in the PD and RA data.

Many modules that contained either cross-talk genes or pyroptosis genes in PD and RA were selected, among which the distribution of cross-talk genes or pyroptosis-related genes was analyzed. The modules containing both cross-talk genes and pyroptosis-related genes were identified and regarded as significant module. The cross-talk genes in the significant modules were considered as core cross-talk genes. In addition, in order to further analyze the functions of significant modules of PD and RA, the functional enrichment analysis of significant module genes was performed by using “clusterProfiler” package (version 3.14) of R program (version 3.6.3) [34]. The enriched functional terms (i.e., GO-BP and KEGG pathway) with *P* value < 0.05 were considered as significant.

### 2.9. Deep Investigation of Core Cross-Talk Genes

The core cross-talk genes identified in the last step were deeply investigated from three aspects: the correlation between the core cross-talk genes and pyroptosis gens in the significant modules; the expression patterns of core cross-talk genes in case and control healthy samples of PD and RA; and the diagnostic accuracy of cross-talk genes by carrying out receiver-operating characteristics (ROC) analysis.

Pearson correlation analysis was performed for the genes obtained in the PD module using the PD disease sample dataset and in the RA module using the RA disease sample dataset. Afterwards, the “ggpubr” package (version: 0.4.0.) of R program (version 3.6.3) was applied to use boxplot to display the expression patterns of core cross-talk genes and pyroptosis-related genes in PD and RA. The Kruskal-Wallis-test was used to analyze the expression values of each gene in disease and control samples. The smaller the *P* value of the result, the greater the difference between case and control samples, and the more “^∗^” is depicted in the boxplots. ROC analysis for the core cross-talk genes was performed to check the diagnostic accuracy of core cross-talk genes at the expression level.

### 2.10. PPI Network Consisted of Core Cross-Talk Genes and Pyroptosis-Related Genes

Based on the gene expression profiles of case samples in PD and RA data, the GENIE3 package (version 3.13) [35] of R program (version 3.6.3) was used to predict the relationship between all genes in the significant modules of PD and RA, as well as cross-talk genes and pyroptosis-related genes. Afterward, the predicted results were ranked by the descending order of the weight value, among which the relationship pairs with the top 10% weight were selected and used as the interaction pairs for constructing the subsequent protein-protein interaction (PPI) network.

The experimentally validated PPIs were obtained from eight databases: HPRD (http://www.hprd.org/index_html), BIOGRID (http://thebiogrid.org/), DIP (http://dip.doe-mbi.ucla.edu/dip/Main.cgi), MINT (http://mint.bio.uniroma2.it/mint/Welcome.do), menthe (http://mentha.uniroma2.it/index.php), PINA (http://cbg.garvan.unsw.edu.au/pina/), InnateDB (http://www.innatedb.com/), and Instruct (http://instruct.yulab.org/index.html). The core cross-talk genes and pyroptosis-related genes in the significant modules of PD and RA were extracted, and these gene-related PPIs were obtained from the eight databases above mentioned. The PPIs obtained from these eight databases and the PPIs obtained by GENIE3 package (version 3.13) were merged together and eventually used as the PPIs used for constructing the significant module-related PPI network. The PPI network was visualized by Cytoscape software (version 3.9.0).

### 2.11. The Relationship between Module Genes and KEGG Pathway

The gene sets of the human KEGG pathway and experimentally validated gene sets of PPI interaction pairs were obtained. These two gene sets were used to observe potential relationships between five important genes (TIMP1, LGALS1, GPX4, VOPP1, and AIM2) and KEGG pathways, based on the gene sets of human KEGG pathways, the pathways in which significant module genes were obtained. After identifying the specific KEGG pathways, all genes that were involved in these pathways were obtained. The relationship between cross-talk genes, KEGG pathways, and pyroptosis-related genes was investigated. Based on the experimentally validated PPI pairs, the respective targets of all genes in each specific pathway in the dataset were obtained. Afterward, if the module genes existed in the targets needs to be checked, the relationship between cross-talk genes, pyroptosis-related genes, and KEGG pathways was shown in table and network.

## 3. Results

### 3.1. Preprocessed Data for the Subsequent Analysis

**Figure 2:**
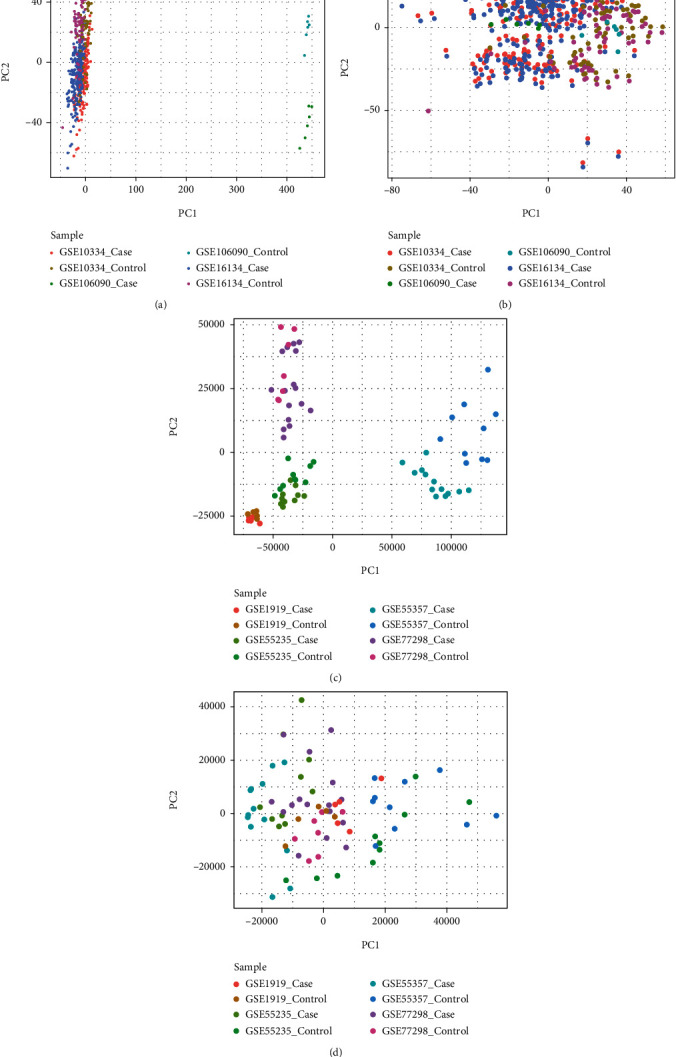
(a, b) PCA analysis results of PD batch before and after correction; (c, d) PCA analysis results of RA batch before and after correction.

After batch correction and normalization of data, a set of PD data and a set of RA data were obtained. The set of PD data contained three datasets (GSE106090, GSE10334, and GSE16134) and consisted of a total of 430 case samples and 139 control samples. The set of RA data contained four datasets (GSE55235, GSE55457, GSE77298, and GSE1919) and consisted of 44 case samples and 32 control samples. Differences among samples were significantly reduced after batch correction (Figures 2(a)–2(d)).

### 3.2. Identification of DEGs Dysregulated in Both Diseases

**Figure 3:**
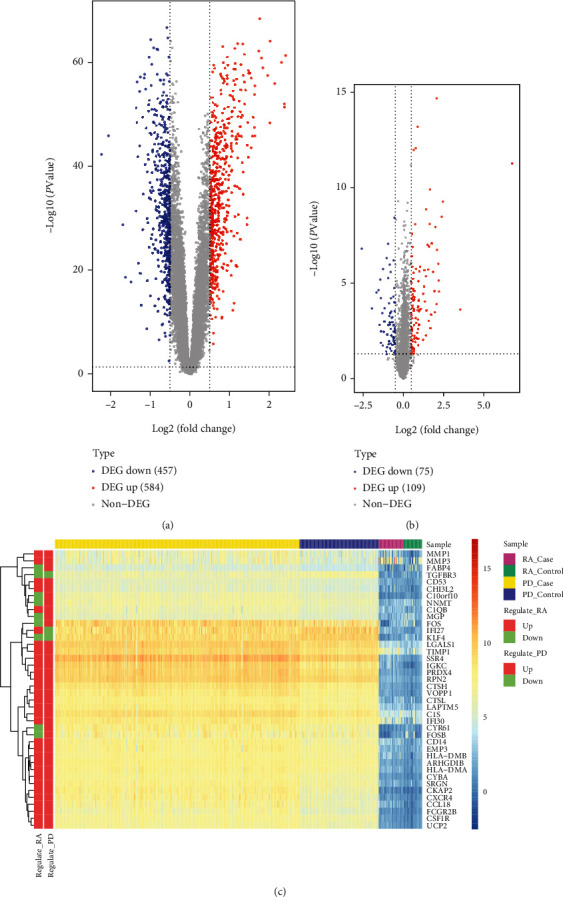
Differentially expressed genes and cross-talk genes. (a) PD differentially expressed gene volcano map; (b) RA differentially expressed gene volcano map; (c) calorimetry of cross-talk gene expression in PD and RA.

The genes with the threshold of *P* value < 0.05 and ∣logFC | > = 0.5 were defined as differential expressed genes (DEGs). The expression pattern of DEGs in both diseases is depicted by using volcano plot (Figures 3(a) and 3(b)). The number of upregulated, downregulated, and the total DEGs is shown in Table 3.

### 3.3. Identification of Cross-Talk Genes

**Figure 4:**
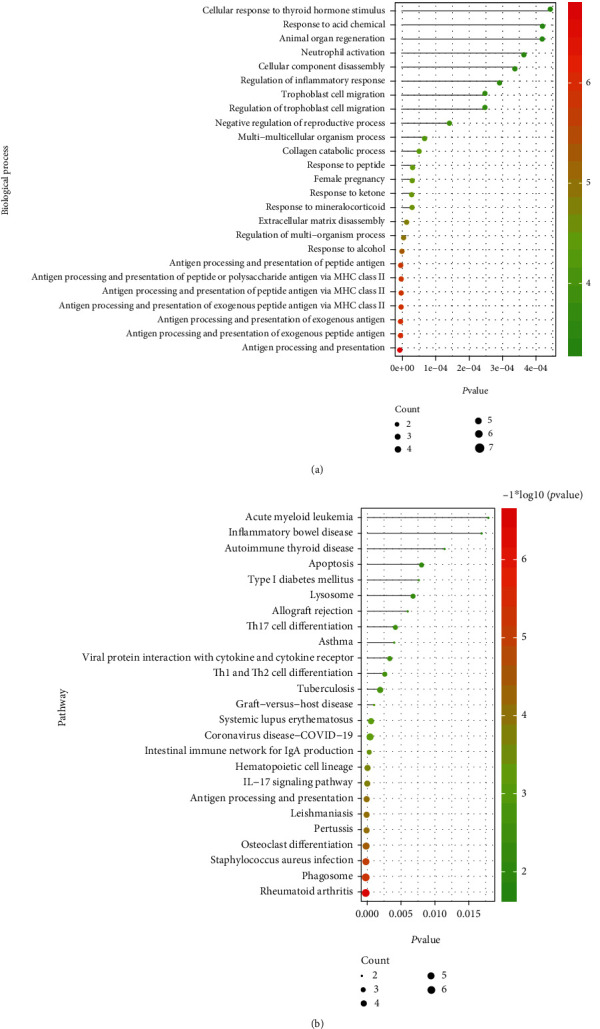
Top 25 significant functions regulated by cross-talk genes. (a) Top 25 biological processes; (b) top 25 KEGG pathway.

By obtaining the intersection of DEGs dysregulated between PD and RA, a total of 40 cross-talk genes were obtained. The expression values of these 40 cross-talk genes in the both sets of PD data and RA data was extracted. The heatmap was therefore plotted to display the expression pattern of 40 cross-talk genes in different samples including RA patients' synovial samples, non-RA subjects' healthy synovial samples, PD patients' inflamed gingival samples, and periodontally healthy subjects' noninflamed healthy gingival samples (Figure 3(c)). Figures 4(a) and 4(b) show the functional terms enriched by the cross-talk genes especially in terms of the biological processes and KEGG signaling pathways.  Figure 4(a) shows that cross-talk genes were mainly enriched in several biological processes, for example, neutrophil activation, extracellular matrix disassembly, and antigen processing and presentation of peptide antigen.  Figure 4(b) shows that cross-talk genes were mainly enriched in several biological processes, for example, apoptosis, lysosome, IL-17 signaling pathway, osteoclast differentiation, and phagosome.

### 3.4. Correlation between Cross-Talk Genes and Pyroptosis-Related Genes

**Figure 5:**
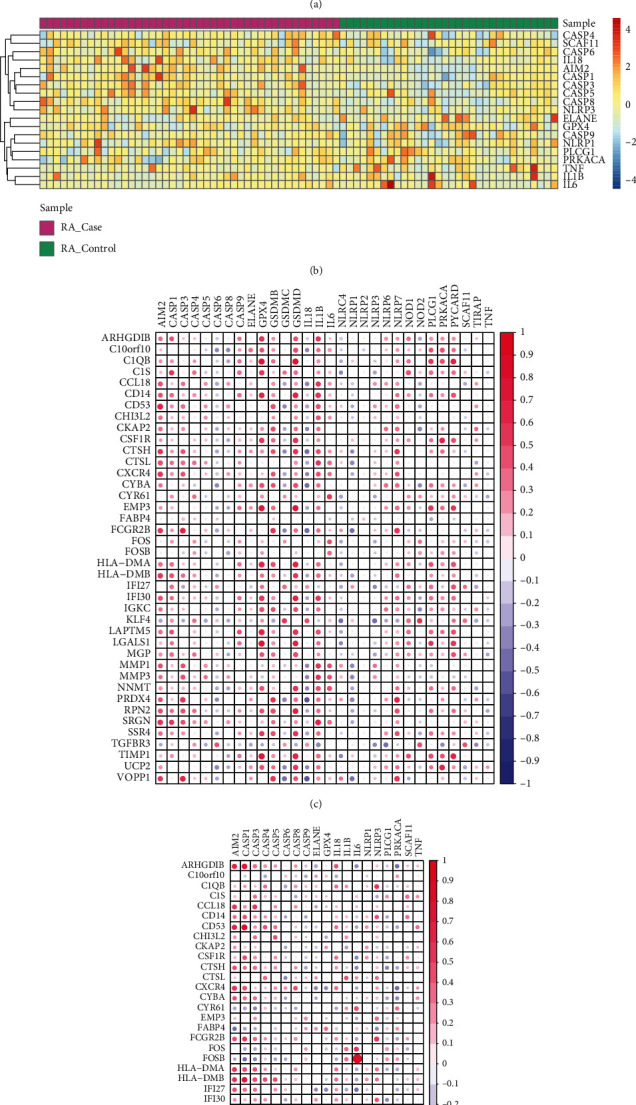
Expression and correlation of pyroptosis-related genes in PD and RA. (a) Heatmap of pyroptosis-related gene expression in PD; (b) heatmap of the expression of pyroptosis-related genes in RA; (c) correlation between cross-talk genes and pyroptosis-related genes in PD samples; (d) correlation between cross-talk genes and pyroptosis-related genes in RA samples.

The expression patterns of 33 pyroptosis-related genes in the sets of PD and RA data were depicted in heatmap (Figures 5(a) and 5(b)). Figures 5(a) and 5(b) showed that majority of the pyroptosis-related genes were not differentially expressed between case and healthy samples, no matter in either PD or RA data. The pyroptosis-related genes (e.g., IL6, AIM2, IL1BIL6, and IL18) were differentially expressed between case and healthy control samples of PD disease, while none of pyroptosis-related genes was differentially expressed between the case and healthy control samples of RA disease. In addition, the gene expression values of pyroptosis-related genes and cross-talk genes in disease samples of PD and RA were obtained, respectively. Therefore, the correlation between 33 pyroptosis-related genes and 40 cross-talk genes were analyzed by Pearson correlation coefficient (Figures 5(c) and 5(d)).

### 3.5. Identification of Common Pathways Involved by Both Cross-Talk Genes and Pyroptosis-Related Genes

**Figure 6:**
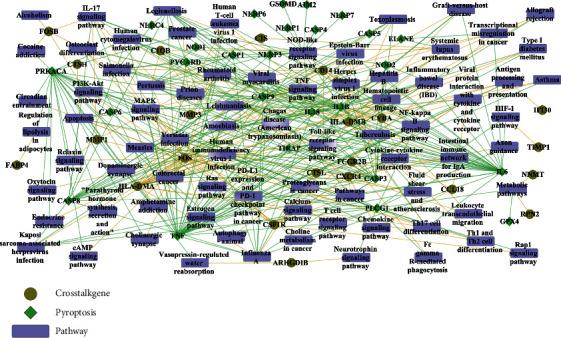
Pathway-gene network, in which cross-talk genes and pyroptosis-related genes are involved.

The human KEGG pathways were obtained from the KEGG database (https://www.kegg.jp/) to identify the pathways in which cross-talk genes interact with pyroptosis-related genes. Cytoscape software was used to construct the pathway-gene network consisted of cross-talk genes and pyroptosis-related genes as well as 77 KEGG pathways (Figure 6). It can be observed from Figure 6 that IL6 and FOSB commonly regulated the IL-17 signaling pathway; CASP1 and HLA-DMB commonly regulated the pathway of Influenza A; three cross-talk genes (FOS, CD14, and CSF1R) together with pyroptosis-related genes (TNF, PRKACA, IL1B, and CASP3) commonly affected the MAPK signaling pathway; CD14 and pyroptosis genes (PLCG1, IL1B, TNF, and TIRAP) commonly regulated the NF-kappa B signaling pathway.

### 3.6. Modules Screened for Cross-Talk Genes and Pyroptosis-Related Genes

**Figure 7:**
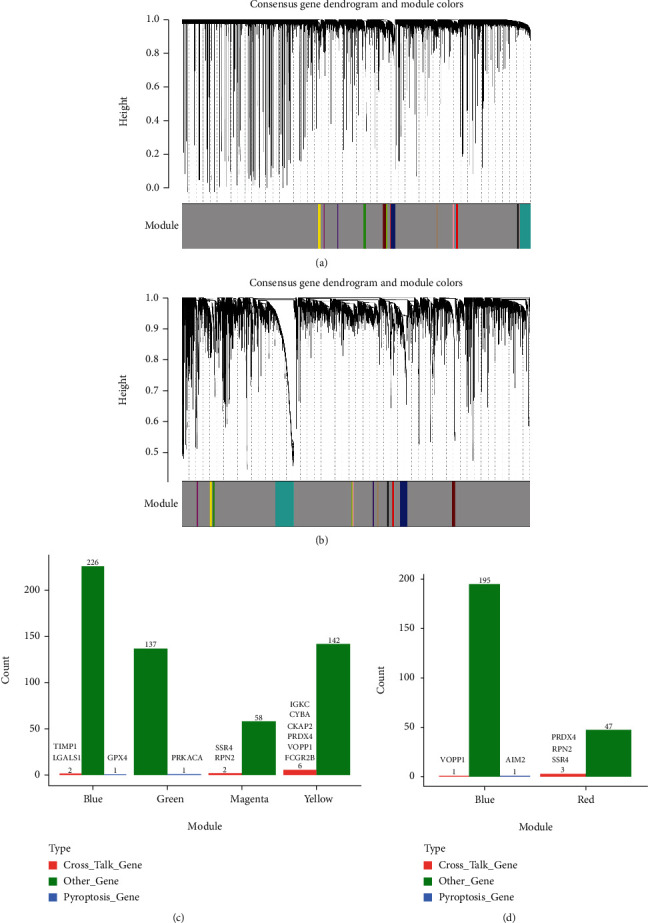
Module screening of cross-talk genes and pyroptosis-related genes. (a, b) WGCNA network module built by PD and RA; (c, d) gene distribution of WGCNA network modules constructed by PD and RA.

In WGCNA network, the *β* value closest to the scale-free network was 14 for PD and 5 for RA. After selecting the *β* value as the network construction parameter, a weighted coexpression network model was established to classify the genes and divide them into several modules. At least 30 genes in each module were set; otherwise, similar gene modules were merged. Module mining was carried out by using WGCNA's cutreeStaticColor method. The coexpression network constructed by PD samples had 15 modules (parameter cutHeight = 0.98, minSize = 30) (Figure 7(a)). The network constructed by RA samples had 13 modules (parameters cutHeight = 0.95, minSize = 30) (Figure 7(b)).

**Figure 8:**
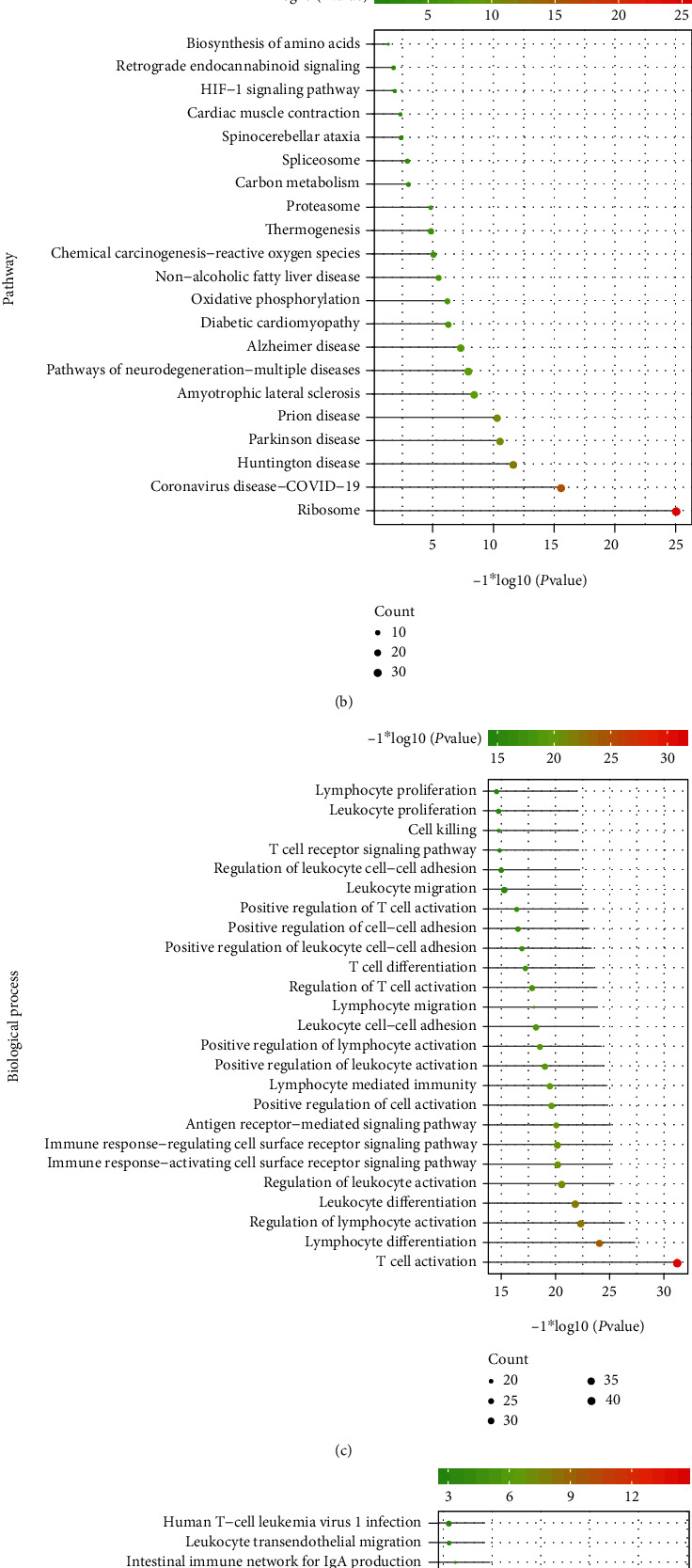
Significant function analysis of significant modules involved in cross-talk genes and pyroptosis-related genes. (a, b) Significant biological processes and pathways of significant module enrichment of PD. GO BP selection top 25 and KEGG pathway selection were all mapped. (c, d) The remarkable biological processes and pathways of module enrichment in RA. Top 25 was selected for mapping both GO BP and KEGG pathway.

The modules that may contain cross-talk genes or pyroptosis-related genes in PD and RA were selected to observe the distribution of cross-talk genes or pyroptosis-related genes in the modules (Figures 7(c) and 7(d)). Both cross-talk genes and pyroptosis-related genes only appear in the blue module of PD and RA, respectively; however, the other modules contained either cross-talk genes or pyroptosis-related genes. Based on this reason, blue module was regarded as the significant module. The functional terms enriched by the blue module genes in PD and RA were, respectively, shown in Figures 8(a)–8(d).  Figure 8(a) shows that blue module genes were significantly enriched in several biological processes involved in PD, for example, protein localization to endoplasmic reticulum, protein targeting to ER, and nucleoside triphosphate metabolic process.  Figure 8(b) shows that blue module genes were significantly enriched in several KEGG pathways involved in PD, for instance, HIF-1 signaling pathway, ribosome, biosynthesis of amino acids, spliceosome, proteasome, and oxidative phosphorylation.  Figure 8(c) shows that blue module genes were significantly enriched in several biological processes, for example, leukocyte proliferation, T cell receptor signaling pathway, antigen receptor-mediated signaling pathway, and immune-response-regulating cell surface receptor signaling pathway.  Figure 8(d) shows that blue module genes were significantly enriched in several KEGG pathways, for example, PD-L1 expression and PD-1 checkpoint pathway, NF-kappa B signaling, B cell receptor signaling, cytokine-cytokine receptor pathway, and natural killer cell-mediated cytotoxicity.

### 3.7. Identification of Core Cross-Talk Genes from the Significant Modules

The blue module was significant in PD and RA networks, respectively. Thus, the cross-talk genes and pyroptosis-related genes in the module were extracted; thereby, three genes, i.e., TIMP1, LGALS1, and GPX4 (pyroptosis), were in the blue module of PD network, while two genes, i.e., VOPP1 and AIM2 (pyroptosis), were in the blue module of RA. Thus, three core cross-talk genes (TIMP1, LGALS1, and VOPP1) were identified. Five genes (TIMP1, LGALS1, GPX4, VOPP1, and AIM2) including three core cross-talk genes and two pyroptosis genes were included for the subsequent analyses.

**Figure 9:**
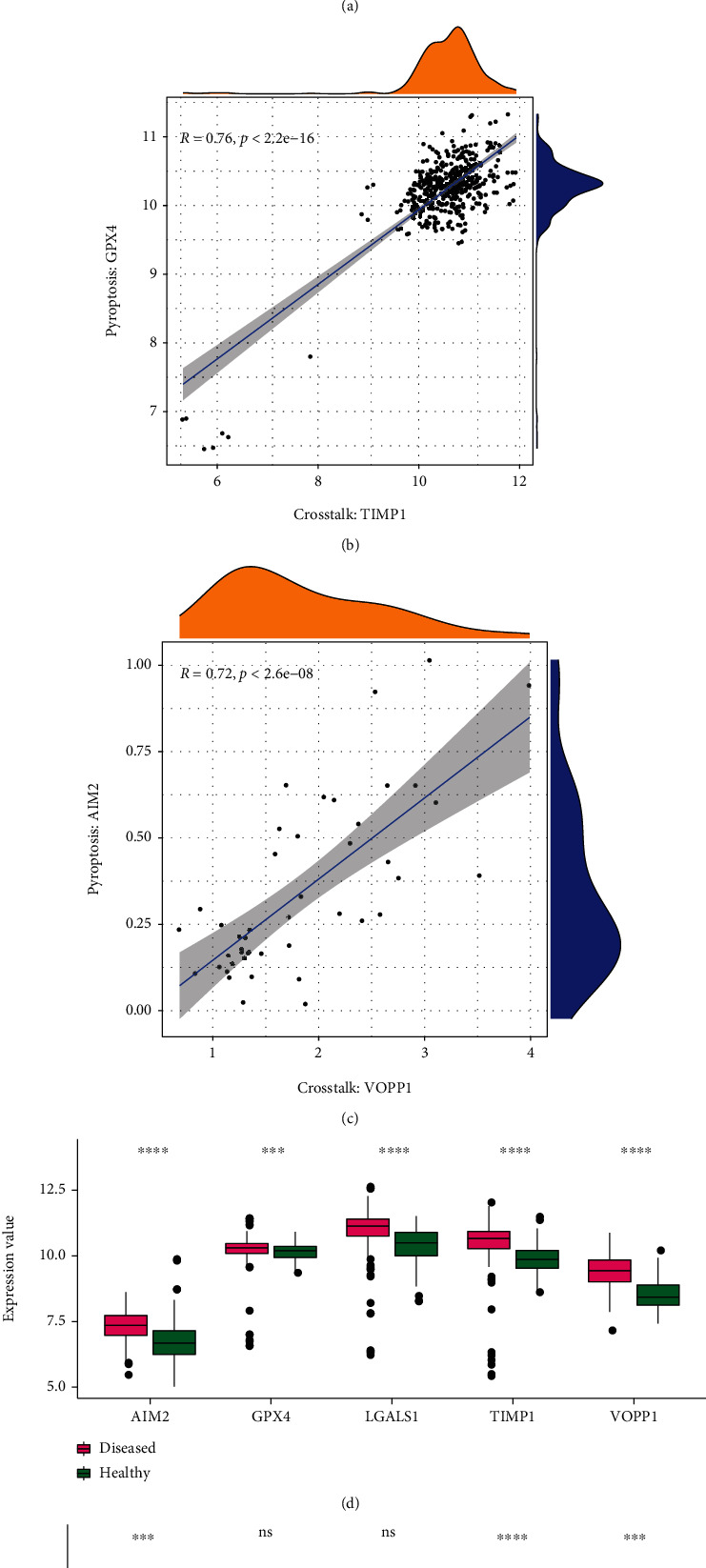
Correlation and predictive analysis of cross-talk genes and pyroptosis-related genes in significant modules of PD and RA. (a) Correlation between LGALS1 and GPX4 in PD samples; (b) correlation between TIMP1 and GPX4 in PD samples; (c) correlation between VOPP1 and AIM2 in RA disease samples. (d) Expression of TIMP1, LGALS1, GPX4, VOPP1, and AIM2 in PD samples and normal samples. (e) Expression of TIMP1, LGALS1, GPX4, VOPP1, and AIM2 in RA and normal samples. *P* values and “^∗^” in the corresponding relation is *P* > 0.05 ns, ^∗^*P* < = 0.05, *P* < ^∗∗^ said = 0.01, *P* < = 0.001, ^∗∗∗^ said^∗∗∗∗^*P* < = 0.0001.

Figures 9(a)–9(c) show the correlation between these three cross-talk genes and the pyroptosis genes in the blue significant module.  Figure 9(a) shows that the correlation between the core cross-talk gene-LGALS1 and pyroptosis gene-GPX4 was strong (*R* = 0.74 > 0.7, *P* < 2.2*e* − 16).  Figure 9(b) shows that the correlation between the core cross-talk gene-TIMP1 and pyroptosis gene-GPX4 was strong (*R* = 0.76, *P* < 2.2*e* − 16).  Figure 9(c) shows that the correlation between the core cross-talk gene-VOPP1 and pyroptosis gene-AIM2 was strong (*R* = 0.72, *P* = 2.6*e* − 08).

Figures 9(d) and 9(e) show the expression patterns of five important genes (IMP1, LGALS1, GPX4, VOPP1, and AIM2) in PD and RA.  Figure 9(d) shows that all these five genes were significantly upregulated in case samples of PD compared with control healthy samples.  Figure 9(e) shows that among five important genes, three genes (AIM2, TIMP1, and VOPP1) were significantly upregulated in case samples of RA, while the other two genes (GPX4 and LGALS1) were not found to be dysregulated in RA.

**Figure 10:**
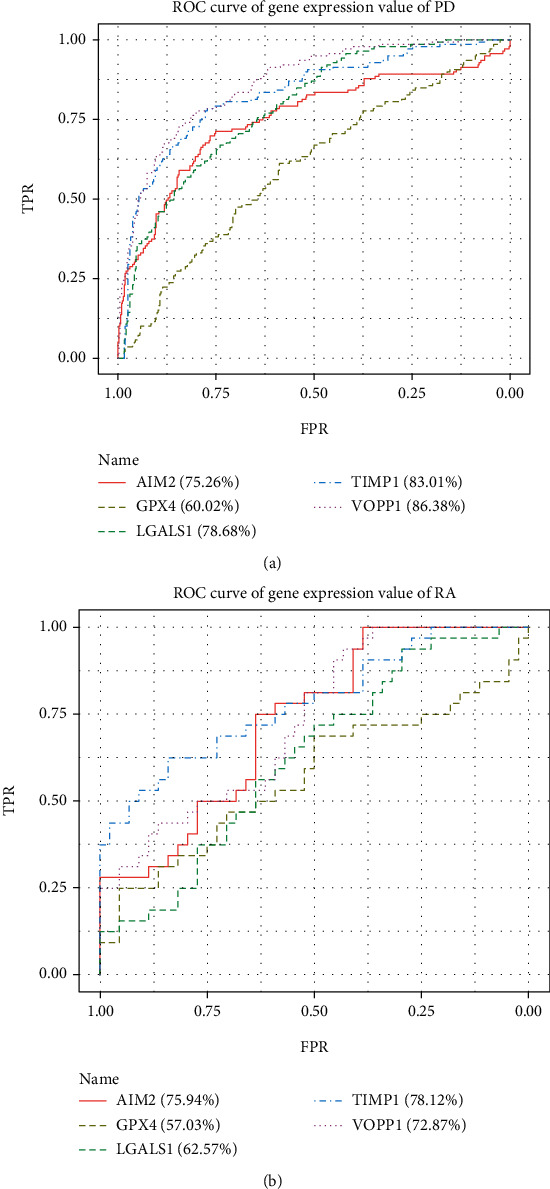
(a) ROC results of TIMP1, LGALS1, GPX4, VOPP1, and AIM2 in PD. (b) ROC results of TIMP1, LGALS1, GPX4, VOPP1, and AIM2 in RA.

Figure 10 used ROC curves to show the diagnostic accuracy of the 5 important genes in PD and RA, respectively. The results showed that three genes (TIMP1, VOPP1, and AIM2) had higher diagnostic accuracy on predicting a specific disease, while the other two genes (LGALS1 and GPX4) showed lower diagnostic accuracy on predicting a specific disease.

### 3.8. Construction of PPI Network of Significant Module

**Figure 11:**
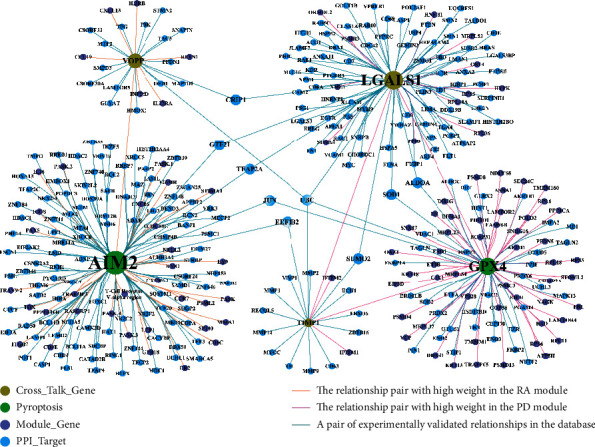
PPI-network of significant module gene related with cross-talk genes and pyroptosis-related genes (IMP1, LGALS1, GPX4, VOPP1, and AIM2).

Based on the expression profile of case samples of PD and RA, the GENIE3 package (version 3.13) of R program (version 3.6.3) was used to predict the weighted relationships between all genes and TIMP1, LGALS1, and GPX4 in the blue module of PD. Meanwhile, all genes in the blue module of RA and the weighted relationships of VOPP1 and AIM2 were predicted in the same manner. Then, PPI relationship pairs related to these five genes (TIMP1, LGALS1, GPX4, VOPP1, and AIM2) were screened from the experimentally validated relationship pairs dataset, and thus, PPI network was constructed and shown in Figure 10. Observed from Figure 11, the interaction pairs (TIMP1-GPX4, LGALS1-GPX4, and VOPP1-AIM2) had higher weight. And also, any two genes among these five important genes can interact with each other by other genes in the PPI network.

### 3.9. The Network Showing the Relationship between Five Important Genes and KEGG Pathways

**Figure 12:**
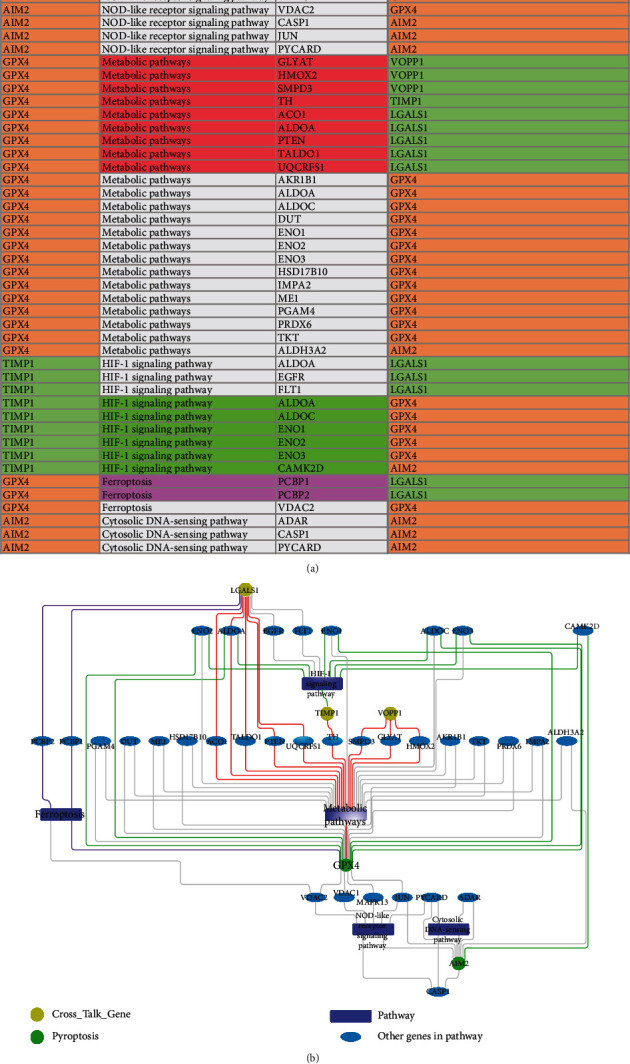
Relationships among TIMP1, LGALS1, GPX4, VOPP1, and AIM2 in related pathways. (a) Table of the relationship between cross-talk genes, pyroptosis-related genes, and pathways. (b) Diagram of the relationship between cross-talk genes, pyroptosis-related genes, and pathways. The background color of (a) is consistent with the color of the lines in (b). The gray line connects the relationship between cross-talk genes or pyroptosis-related genes of the same type. The other color lines connect the relationship between cross-talk genes and pyroptosis-related genes.

Figure 12 used table and network to show the relationship between five important genes and KEGG pathways. Observed from Figure 12, the pyroptosis gene-GPX4 and cross-talk gene-LGALS1 commonly regulated the metabolic pathway. The cross-talk gene-LGALS1 can indirectly regulate ferroptosis by interacting with PCBP2. The cross-talk gene-TIMP1 was involved in the regulation process of HIF-1 signaling pathway. The cross-talk gene-TIMP1 was able to indirectly influence the metabolic pathway by interacting with TH gene. VOPP1 was found to indirectly affect the metabolic pathways by interacting with metabolic pathway-involved genes (e.g., SMPD3, GLYAT, and HMOX2). The pyroptosis-related gene-AIM2 was found to mainly regulate two pathways: cytosolic DNA-sensing pathway and NOD-like receptor signaling pathway. GPX4 was found to indirectly influence AIM2-regulated pathways based on the interaction between GPX4 and JUN, as well as the interaction between JUN and AIM2. AIM2 was found to indirectly regulate the HIF-1 signaling pathway by interacting with CAMK2D gene. The cross-talk gene-TIMP1 was found to be able to regulate the HIF-1 signaling pathway.

## 4. Discussion

A total of five modular genes, i.e., TIMP1, LGALS1, GPX4 (PD network), VOPP1, and AIM2 (RA network) were revealed, of which TIMP1, VOPP1, and AIM2 had the highest predictive effect. These cross-talk or pyroptosis-related genes were found to be involved in the HIF-1 signaling pathway, ferroptosis, metabolic pathways, NOD-like receptor signaling pathway, and cytosolic DNA-sensing pathway. Based on the high variety of results in this current analysis, the discussion will focus on these main findings.

In general, PD and RA are closely related to each other, which has been repeatedly examined during the past years. A recent meta-analysis revealed that patients with PD have a 69% greater risk of having RA than healthy controls [1]. Accordingly, different biologic mechanisms were found to be involved in the complex interrelationship between the two diseases [36]. The most popular and relevant link appears to be within dysbiosis of the oral microbiome; a recent study found that anti-CCP-positive patients show a dysciotic subgingival microbiome [37]. In this context, *Porphyromonas gingivalis* was found to be a key player by causing an immunological imbalance, which promotes RA development and severity [38]. However, the complexity of RA pathogenesis and, especially, the variety of antirheumatic medications affect the periodontal outcome [39]. Accordingly, the identification of potential cross-talk genes appears reasonable to gain a deeper understanding of the interplay between the two diseases. Especially, up until now, there is no information on the potential relevance of pyroptosis and potentially relevant mechanisms in this context. The following will focus on this question and will discuss the potential pathways linking PD and RA in context of pyroptosis and genetic cross-talk.

Primarily, TIMP1 was found to present the highest predictive value in both, PD and RA. TIMP1, i.e., the tissue inhibitor of matrix metalloproteinases (MMP), is a natural antagonist of MMP and thus a glycoprotein with the ability to inhibit matrix degradation [40]. Because matrix degradation, which is triggered by MMP, is a relevant process in hard and soft tissue destruction in both RA and PD [40, 41], the potential role of TIMP1 seems plausible. Different clinical studies examined TIMP1 in patients with RA and PD, whereby both saliva and blood has been analyzed. While the overall results across studies are inconsistent, a clinical study on patients with RA under methotrexate therapy revealed increased TIMP1 and MMP8 concentrations in blood of individuals with RA and periodontal inflammation, indicating an immunologic dysbalance of these patients [42]. In the current study, TIMP1 was found to be related to the HIF1 signaling pathway. Hypoxia-inducible factor 1 (HIF1) is associated with autoimmune inflammation, because ongoing immune response is oxygen-consuming, leading to an “inflammatory hypoxia” of respective affected environments [43]. Regarding PD, HIF1 can be related to mucosal tissue aging [44], apoptotic-osteocyte-mediated osteoclastogenesis [45], and apoptosis and autophagic cell death in human periodontal ligament cells [46] as well as inflammatory reaction on periodontal ligament cells triggered by *Porphyromonas gingivalis* [47]. Due to the potential role of *Porphyromonas gingivalis* in RA pathogenesis, the latter could be of particular interest [4]. Similarly, as for PD, hypoxia and the HIF1 pathway are also related to RA, especially with regard of the vascular angiogenesis of the synovial membrane [48]. Accordingly, an immunological dysbalance by a deregulation of TIMP1 alongside with a role of hypoxia and HIF1 pathway could be relevant interlinks between PD and RA.

While TIMP1 affected the HIF1 pathway, another gene, i.e., AIM2 as pyroptosis-related gene indirectly influenced the HIF1 pathway. AIM2 (absent in melanoma 2) is a cytoplasmic sensor of DNA originating from destructed cellular structures or pathogens, respectively [49]. A genome-wide association study confirmed AIM2 to be a risk gene for periodontitis development [50]. Furthermore, polymorphisms of AIM2 were found to be related to higher levels of periodontal microorganisms and worse periodontal parameters, indicating a role in PD [51]. Thereby, *Porphyromonas gingivalis* could be involved as well, because this bacterium was found to induce IL-1*β* secretion and pyroptotic cell death related to AIM2 inflammasome activation [52]. Therefore, the potential role of pyroptosis and related genes in the interplay between RA and PD seems probable. The second pyroptosis-related gene, i.e., GPX4, was involved in metabolic pathway, ferroptosis, and NOD-like receptor pathway and interacts with JUN, which in turn interacts with AIM2. Therefore, GPX4 also potentially affects the pathway regulated by AIM2 through interaction. GPX4 (glutathione peroxidase 4) is a selenoperoxidase, which is decisively involved in antiperoxidant defense and related to ferroptosis [53]. GPX4 was not examined regarding periodontitis, yet; however, glutathione peroxidases in general are related to periodontal inflammation and thereby related to PD-associated oxidative stress [54, 55]. Additionally, a potential role of ferroptosis in periodontal inflammation has been discussed [56]. Similarly, in case of lipopolysaccharide-induced synovitis, GPX4 is reduced, while ferroptosis is triggered [57]. Furthermore, as oxidative stress occurs in autoimmune rheumatic disease, glutathione peroxidase in general appears of relevance in these diseases [58].

Two further genes, i.e., LGALS1 and VOPP1, were also found as module-related genes. LGALS1, i.e., galectin-1, is a *β*-galcotosid-binding lectin, which is able to induce T cell apoptosis and is also involved in modulation of inflammation [59]. Galectin-1 was found to be increased in gingival crevicular fluid of PD individuals, suggesting its potential role in periodontal inflammation [60]. Interestingly, soluble galectin-1 can bind to lipopolysaccharides in the cell wall of *Porphyromonas gingivalis*, enhancing the adhesion and invasion of this bacterium into oral epithelial cells [61]. Intracellular galectin-1 was able to inhibit lipopolysaccharide-induced autophagy and apoptosis in periodontal ligament cells [62]. This is a further hint of the potential role of *Porphyromonas gingivalis* and its lipopolysaccharides in the interrelationship between PD and RA. Furthermore, galectin-1 was also found to be of relevance in the pathogenesis of RA; thereby, RA patients show higher levels of galectin-1, what is positively correlated to disease activity [63]. Lastly, vesicular overexpressed in cancer prosurvival protein 1 (VOPP1) was deregulated in the current study, which was found the be related to oxidative cellular injury [64]. While this gene is related to different cancers, including squamous cell carcinoma, hepatocellular carcinoma, or breast cancer [64–66], no results regarding PD or RA are available. However, the potential relevance in oxidative stress could support the relevance of this issue as mentioned above.

Altogether, several hypotheses can be formed based on the current study's results: (I) oxidative stress and hypoxia might be a common condition in PD and RA and thus linking its pathogenesis; (II) *Porphyromonas gingivalis* and its virulence factors repeatedly occur in the context of the modular genes, underlining the potential of a microbiological interlink; (III) pyroptosis, including the pyroptosis-related genes AIM2 and GPX4, appears to be involved in the interrelationship between PD and RA. All these hypotheses need experimental validation. Therefore, the current study provides a basis for future research in the field.

### 4.1. Strengths and Limitations

This is the first bioinformatics study on PD and RA under consideration of pyroptosis-related genes. The analysis was very comprehensive and revealed a variety of results, which can be a basis for future research. A good number of samples, which underwent a correction within the two disease groups strengthens the findings. The major limitation is that the results are only based on bioinformatics analysis; thus, an experimental validation of the hypotheses must be performed subsequently. It must be considered that the RA and PD patients were different and heterogeneous groups of individuals. Thereby, disease-related or therapy conditions (e.g., RA medication) were not considered, because only data are analyzed. Generally, the findings are on transcriptomic level, what must be recognized during interpretation of the findings.

## 5. Conclusion

The current study showed shared mechanisms between PD and RA via cross-talk and pyroptosis genes and related pathways, supporting the close interrelationship between RA and PD. Thereby, five modular genes, i.e., TIMP1, LGALS1, GPX4, VOPP1, and AIM2, were revealed, which could be of relevance in the interlink between RA and PD and might serve as potential biomarkers. These findings are a basis for future research in the field.

## Figures and Tables

**Table 1:** The information of included datasets.

Disease	Series	Sample type	Experimental type	Platform	Case	Control
Rheumatoid arthritis	GSE55235	Synovial tissue	Microarray	GPL96	10	10
	GSE55457	Synovial tissue	Microarray	GPL96	13	10
	GSE77298	Synovial biopsies	Microarray	GPL570	16	7
	GSE1919	Synovial tissue	Microarray	GPL91	5	5
Periodontitis	GSE16134	Gingival tissue	Microarray	GPL570	241	69
	GSE10334	Gingival tissue	Microarray	GPL570	183	64
	GSE106090	Gingival tissue	Microarray	GPL21827	6	6

**Table 2:** Pyroptosis-related genes from literature.

Genes	Full-names
AIM2	Absent in melanoma 2
CASP1	Cysteine-aspartic acid protease-1
CASP3	Cysteine-aspartic acid protease-3
CASP4	Cysteine-aspartic acid protease-4
CASP5	Cysteine-aspartic acid protease-5
CASP6	Cysteine-aspartic acid protease-6
CASP8	Cysteine-aspartic acid protease-8
CASP9	Cysteine-aspartic acid protease-9
ELANE	Elastase, neutrophil expressed
GPX4	Glutathione peroxidase 4
GSDMA	Gasdermin A
GSDMB	Gasdermin B
GSDMC	Gasdermin C
GSDMD	Gasdermin D
GSDME	Gasdermin E
IL18	Interleukin 18
IL1B	Interleukin 1 beta
IL6	Interleukin 6
NLRC4	NLR family CARD domain containing 4
NLRP1	NLR family pyrin domain containing 1
NLRP2	NLR family pyrin domain containing 2
NLRP3	NLR family pyrin domain containing 3
NLRP6	NLR family pyrin domain containing 6
NLRP7	NLR family pyrin domain containing 7
NOD1	Nucleotide binding oligomerization domain containing 1
NOD2	Nucleotide binding oligomerization domain containing 2
PJVK	Pejvakin/deafness, autosomal recessive 59
PLCG1	Phospholipase C gamma 1
PRKACA	Protein kinase cAMP-activated catalytic subunit alpha
PYCARD	PYD and CARD domain containing
SCAF11	SR-related CTD-associated factor 11
TIRAP	TIR domain containing adaptor protein
TNF	Tumor necrosis factor

**Table 3:** Statistics of the number of differentially expressed genes.

Disease	Up-DEG	Down-DEG	Total-DEG
PD	584	457	1041
RA	109	75	184

## Data Availability

The datasets used and/or analyzed during the current study are available from the corresponding author on reasonable request.

## Supplementary Materials

Supplementary Materials Table S1: the detailed clinical sample information of each dataset analyzed in the current research.
